# Risk perception, attitudes, and quality of life in a multicomponent benzodiazepine deprescription strategy

**DOI:** 10.1016/j.rcsop.2025.100666

**Published:** 2025-09-30

**Authors:** Ingrid Ferrer López, Antonio Olry de Labry-Lima, Alicia Gutiérrez-Valencia, Encarnación García Bermúdez, Francisco Atienza Martín, Amalia García-Delgado Morente, María Dolores Murillo Fernández, Yolanda Sánchez Cañete, Clara Bermúdez-Tamayo

**Affiliations:** a Primary Care District, Primary Care Clinical Management Unit. Andalusian Health Service. Seville, Spain; b Andalusian School of Public Health. Granada, Spain.; c Ciber de Epidemiología y Salud Pública- CIBERESP. Barcelona, Spain; d Ibs.Granada. Instituto Biosanitario de Granada. Granada, Spain; e Primary Care District, Condado-Campiña. Primary Care Clinical Management Unit, Andalusian Health Service. Huelva, Spain; f Royal and Illustrious Official College of Pharmacists of Seville. Seville, Spain; g Primary Care District, Clinical Management Unit El Porvenir. Andalusian Health Service. Seville, Spain; h Community Pharmacy. Morente García-Delgado. Seville, Spain; i Community Pharmacy. Fernández Vega. Seville, Spain; j Primary Care District, Clinical Management Unit Amante Laffón. Andalusian Health Service. Seville, Spain; k Universidad de Granada. Granada, Spain

**Keywords:** Benzodiazepine, Deprescription, Inappropriate prescribing, Prescription drug overuse

## Abstract

**Background:**

Multicomponent strategies can reduce benzodiazepine (BZD) use. BenzoStopJuntos (Spanish for “Stop Benzos Together”), a multidisciplinary deprescribing programme of the Andalusian Health Service, supports patients to taper/stop BZD through education, behavioral support, and non-pharmacological alternatives. We evaluated whether early changes (6 months) in risk perception and attitudes—and secondarily, quality of life—were associated with long-term discontinuation of BZD.

**Methods:**

In a quasi-experimental pre–post study in two primary care centres (Seville, Spain; *n* = 243), the intervention included patient education, tapering support, and alternatives for anxiety/insomnia delivered by a multidisciplinary team. Primary outcomes were (a) short-term (6-month) changes in risk perception and attitudes and (b) long-term BZD discontinuation over 5.5 years; the secondary outcome was quality of life (WONCA/COOP), monitored to detect potential harms. Multivariable logistic regression examined whether 6-month changes in beliefs/attitudes predicted subsequent discontinuation, adjusting for sociodemographic and clinical factors.

**Results:**

BZD discontinuation increased from 31.3 % at 6 months to 40.7 % at 5.5 years. Participants who considered BZD safe long-term were more likely to continue use (OR = 2.0; 95 % CI: 1.6–2.6). Fears of worsened anxiety/sleep strongly predicted persistence (OR = 4.7; 95 % CI: 3.6–6.1). Prior intermittent vs continuous use favored discontinuation (OR = 4.9; 95 % CI: 3.7–6.5). Quality of life improved in emotional, social, and physical domains, with no deterioration observed during follow-up.

**Conclusions:**

Tailored education and behavioral strategies changed risk perceptions and attitudes, which in turn facilitated sustained BZD discontinuation without adverse effects on quality of life. Addressing patient beliefs and encouraging intermittent use patterns may enhance deprescribing success.

**Trial registration:**

ClinicalTrials.govNCT06209827

## 1 Background

Benzodiazepines (BZD) act on the central nervous system, exerting hypnosedative, anxiolytic, and anticonvulsant effects. *Z*-drugs, although not BZD, have similar hypnotic properties and are prescribed for the short-term treatment of insomnia.^1^ Despite clinical guidelines recommending short-term use of these medications, prolonged consumption is prevalent globally,^2^^,^^3^ posing significant health risks. Spain reports the highest per capita consumption of BZD worldwide, with rates steadily increasing over recent years.^4^ Notably, between 2019 and 2022, the defined daily dose (DDD) per 1000 inhabitants per day (DDD/1000/day) for these medications increased from 87.9 to 93.3, representing a 6.1 % growth. This escalation is particularly evident among women and individuals aged 75 years and older.^5^^,^^6^ Andalusia stands out with a consumption rate exceeding the national average across all drug categories, highlighting the challenges in the region with respect to the overuse of BZD.^5^^,^^7^

The COVID-19 pandemic likely contributed to the rising consumption of BZD, as increased anxiety, depression, insomnia, and psychological distress led to higher prescription rates, especially among older adults and those with pre-existing mental health conditions. This surge may have hindered deprescribing efforts and increased dependence.^8^

Prolonged use is associated with tolerance, dependence and significant health risks, including an increased likelihood of falls, cognitive impairment, functional decline, avoidable hospitalizations and mortality, particularly among older adults.^9^ Given these safety concerns and high consumption rates, deprescribing BZD has become a clinical priority for several health organizations. Initiatives such as Choosing Wisely in the United States and Canada promote informed discussions to reduce unnecessary treatments.^10^ Additionally, the National Institute for Health and Care Excellence (NICE) in the United Kingdom develops evidence-based guidelines for safer prescribing,^11^ while GuíaSalud in Spain,^12^ led by the Ministry of Health and supported by scientific societies, facilitates the implementation of clinical practice guidelines to optimize rational medication use.

Numerous studies have examined the factors influencing the deprescribing of BZD and *Z*-drugs, identifying several aspects that impact this process^13^^,^^14^ encompassing system-level factors, provider-related elements, and patient-related factors. Within the patient domain, critical aspects include attitudes and beliefs about the consequences of discontinuing medication, treatment knowledge, trust in healthcare providers, and patient characteristics.^15^

In response to the ongoing challenge of BZD overuse, the Andalusian Health Service in Spain introduced *BenzoStopJuntos*, a multidisciplinary intervention designed to educate and empower patients to reduce and discontinue BZD use. This programme integrates patient education and alternative treatment strategies to support deprescribing. Since patients' attitudes—such as their beliefs about the long-term safety of BZD and concerns about worsening anxiety or sleep upon discontinuation—are known to be significant barriers,^16^ understanding how these attitudes shift during the intervention is critical. Additionally, improvements in health status during the early stages of the programme, particularly within the first six months, may play a key role in enabling patients to successfully quit BZD. The aim of this study was to evaluate whether change in risk perceptions and attitudes toward BZD use during the first six months of the *BenzoStopJuntos* intervention are associated with successful BZD discontinuation without a deterioration in quality of life.

## 2 Methods

This study was designed and reported following the SPIRIT (Standard Protocol Items: Recommendations for Interventional Trials) guidelines.^17^

### 2.1 Design and study population

A quasi-experimental pre-post study to evaluate whether changes in risk perception, attitudes and health status during the first six months of the *BenzoStopJuntos* intervention are associated with successful BZD discontinuation. This design allows the assessment of intervention impact under real-world conditions.

The study was conducted in two primary care centres located in Seville, Spain. The Primary Care District Seville was chosen as the study setting due to its diverse demographic characteristics, including both urban and semiurban populations, providing a representative sample of patients typically seen in primary care for long-term BZD use. The inclusion of Primary Care District also reflects their existing infrastructure and experience in managing multidisciplinary interventions, which facilitated the implementation of the *BenzoStopJuntos* programme.

Participants were recruited from the patient populations served by the two centres involved in the study during the first half of 2018. Recruitment was conducted by a healthcare team, including general practitioner, nurses, and community pharmacists, operating in the area.

The healthcare team members operated at the same sites and coordinated recruitment efforts through scheduled meetings and shared records to prevent duplication. External health professionals, such as primary care pharmacists, participated in follow-up and monitoring activities but were not involved in the recruitment process. All team members, except for the community pharmacist, had access to relevant patient medical data for both recruitment and follow-up. The community pharmacist only had access to patients' medication history. Meetings and telephone follow-ups, led by the primary care pharmacist and the drug information officer from the College of Pharmacists, aimed to standardize the intervention, align objectives, improve data collection, and enhance communication and teamwork.

Eligible participants were adults (>18 years) who had been prescribed BZD and met the inclusion criteria as follows: users of health centres with more than 4 weeks of BZD use, without severe mental disorders, nonterminal, without alcohol dependence or dementia. The exclusion criteria also included intellectual disabilities or any conditions impeding their ability to complete self-report questionnaires. Participants were excluded from the analysis if baseline and 6-month follow-up data were non-completed.

The initial data collection was carried out by health professionals using a self-administered questionnaire completed by patients. At 6 months, data were collected by external health professionals via telephone interviews to minimize bias due to familiarity with participants. Dispensing and sociodemographic data were retrieved from the dispensing database of the Andalusian Health Service.

The study was registered at ClinicalTrials.gov NCT06209827 and the full trial protocol is available upon request.

### 2.2 Intervention

The BenzoStopJuntos programme was delivered in routine primary care by general practitioners, nurses, social workers, and community/primary-care pharmacists. It combined patient education, a standardized but individualised tapering protocol, endorsed written materials, and non-pharmacological supports. Multiple components were included as follows, on the basis of previous studies.^18^, ^19^, ^20^, ^21^

a) Educational Session: Healthcare professionals engaged participants in an informative discussion covering the risks and benefits of BZD use and possible non-pharmacological alternatives. This discussion addressed healthcare providers' concerns regarding prolonged BZD use and presented practical solutions. Patients were provided with an educational brochure, adapted from Canadian Desprescribing Network materials, which included a visual dose-tapering guide to mitigate withdrawal symptoms. The adaptation ensured relevance to the Spanish context.
b) Optional Medical Consultation: If needed, participants were offered a brief consultation with a general practitioner to discuss dose reduction options. Participants if necessary were also referred to a social-educational group to support behavioral change and coping strategies.
c) Supportive Messaging: A brochure featuring a letter endorsed by six scientific societies was included to reinforce the intervention's credibility. Evidence shows that the involvement of these organizations acts as an enabling factor in behavioral change interventions such as this one^22^^,^^23^ The letter explained the risks associated with long-term BZD use and how the professionals were concerned about the chronic use BZD regimen,^20^ potential side effects of prolonged use.^24^ Patients were encouraged to consult further with healthcare professionals about their BZD use.
d) Alternatives to BZD Use: Participants were informed about non-pharmacological options, such as cognitive behavioral therapy (CBT) and self-help resources for managing anxiety and insomnia, to support them through the tapering process.

A standardized tapering regimen was proposed as the initial approach for all participants. The duration of dose reduction, however, was tailored to each patient's needs. In individuals with a high degree of dependence, the tapering was further extended during the final weeks to prevent withdrawal symptoms. A detailed description of the tapering protocol and all components of the intervention has been previously published.^25^

### 2.3 Outcomes, follow-up, and measurement methods

Three validated questionnaires were used in this study: (1) a risk perception and attitudes questionnaire based on the EMPOWER study,^26^ (2) the WONCA/COOP quality of life instrument,^27^ and (3) a clinical data collection form incorporating the Charlson Comorbidity Index.^28^ All three questionnaires were pilot-tested in a sample of the study population to ensure clarity and feasibility.

The primary outcome was successful BZD discontinuation, assessed at 6 months and sustained at 5.5 years, defined as self-reported cessation corroborated by the absence of BZD dispensations during the relevant periods. Secondary outcomes were 6-month changes in risk perception and attitudes toward BZD use (EMPOWER-based questionnaire) and quality of life (WONCA/COOP), plus healthcare resource utilization.

For the first five domains, scores range from 1 to 5 are categorized as low-moderate (1–3) or high (4–5). Overall health is classified as poor (1–3) or good (4–5), while changes in health are categorized as declined health (1–3) or stable/improved health (4–5).

Additional data collected included BZD dispensing records, with non-dispensing defined as the absence of BZD dispensing during the last two periods of each analysis period. Other collected variables included BZD indications, attendance at mental health consultations, the Charlson comorbidity index (a measure of comorbidity affecting 10-year survival), and the duration of BZD use.

**Fig. 1:**
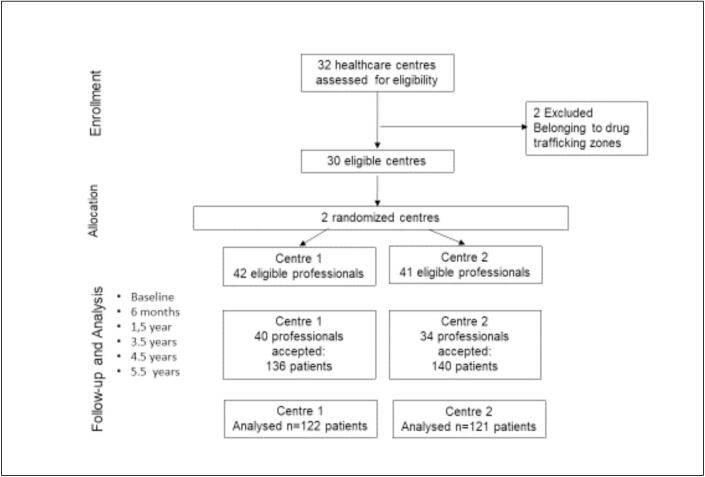
Participant flow and follow-up points. Centre 1 – participating professionals (*n* = 40): 14 general practitioners, 14 nurses, 11 pharmacists, 1 social worker. Centre 2 – participating professionals (*n* = 34): 7 general practitioners, 13 nurses, 9 pharmacists, 1 social worker.

The timeline of participant assessments of BZD use was structured to capture both short-term and long-term outcomes. Data on BZD use, risk perceptions and attitudes about their use, and health status were collected for all participants at baseline and 6 months after intervention. This period was used as a control to compare the effects of the intervention. Assessments were conducted at baseline, followed by subsequent evaluations at 6 months, 1.5 years, 3.5 years, 4.5 years, and 5.5 years post-intervention. A detailed diagram of participant flow and follow-up points is presented in Fig. 1.

### 2.4 Sample

The sample size was calculated to detect a 15 % increase in BZD discontinuation rates post-intervention, based on prior studies of similar interventions.^29^ A sample of 230 participants was required to achieve 80 % power with a significance level of 0.05. To account for an anticipated 20 % drop-out rate over the follow-up period, 276 participants were recruited, ensuring sufficient power despite attrition.

Initially, 32 healthcare centres were evaluated for eligibility, with two centres excluded due to their location in drug trafficking zones, resulting in 30 eligible centres. Two centres were selected by computer-generated simple random sampling without replacement, using a random sequence prepared by an independent researcher not involved in recruitment, intervention delivery, or analysis; centre selection was concealed from clinical teams until invitations were issued.

In total, 276 patients were initially recruited during their regular clinical practice across both centres. However, due to incomplete data at baseline and the 6-month follow-up, some patients were excluded from the final analysis. Centre 1 retained 122 patients, with 40 participating professionals 14 general practitioners, 14 nurses, 11 pharmacists, and 1 social workers across the health centres, while Centre 2 retained 121 patients, with 34 professionals involved. 7 general practitioners, 13 nurses, 9 pharmacists, and 1 social workers across the health centres.

Clinicians (GPs, nurses, pharmacists, social workers) recruited participants and delivered the intervention in routine care. Patients self-completed baseline questionnaires in clinic; 6-month outcomes were collected by external staff via telephone; long-term discontinuation was ascertained from the administrative dispensing database. Clinicians did not extract or analyse study data.

### 2.5 Statistical analysis

The baseline characteristics of the participants are presented as numbers and percentages for categorical variables and means with standard deviations for continuous variables. McNemar's test was applied to compare changes in risk perceptions, attitudes and health status from baseline to 6 months after intervention for categorical variables. The changes in BZD use over time were evaluated via McNemar's test to assess the differences in the proportions of participants who continued or discontinued BZD use at each time point. To assess the overall trend in BZD use across all time points, Cochran's Q test was applied. This nonparametric test is used to determine whether the proportion of participants who continue BZD use changes significantly across multiple time points, making it appropriate for analyzing longitudinal data where the same participants are assessed repeatedly.

For the longitudinal analysis, the data were reshaped from wide to long formats, accounting for repeated measures across time points. For the association analyses, the dependent variable was sustained BZD discontinuation at 5.5 years (primary outcome). Independent variables were 6-month changes in risk perception, attitudes, and quality of life (secondary outcomes), with models adjusted for prespecified covariates. Logistic regression models were used to estimate adjusted odds ratios (ORs) with 95 % confidence intervals (CIs), assessing the likelihood of continued benzodiazepine (BZD) use after 5.5 years on the basis of changes in risk perception, attitudes and health status observed at 6 months. The models controlled for individual characteristics such as mental health status, gender, healthcare centre, age, income, and comorbidity. Our primary objective was to evaluate changes over time rather than to model individual participant trajectories. By adjusting the models for baseline covariates and controlling for potential confounders, we obtained estimates of the intervention's effects.

A *p*-value of less than 0.05 was considered to indicate statistical significance. Collinearity was assessed, and a high degree of multicollinearity was identified between the 4.5-year follow-up and the adjacent 3.5- and 5.5-year follow-ups. Including the 4.5-year time point compromised estimate stability and inflated standard errors; therefore, we excluded the 4.5-year follow-up from the adjusted models. Goodness-of-fit for the logistic regression models was assessed using likelihood ratio tests and Akaike Information Criterion (AIC). Model comparisons were conducted to ensure appropriate model specification and to evaluate the improvement in model fit after adjusting for potential confounders.

Missing values for the primary and secondary outcomes were imputed via multiple imputation via the chained equations method. This method assumes that data are missing at random and generates multiple datasets where the missing values are replaced by plausible estimates on the basis of observed data. We generated 20 imputed datasets, ensuring robust estimation of missing values. Convergence was assessed through trace plots, and diagnostic checks confirmed the adequacy of the imputation process. Sensitivity analyses were conducted to compare the results from the imputed data with those from a complete case analysis, ensuring consistency across approaches.

## 3 Results

### 3.1 Participant characteristics

Two hundred seventy-six participants were recruited, and two hundred forty-three participants were analysed. Six participants (2.5 %) died during the 5.5 years of follow-up. The majority of participants were women (69.5 %). The mean age of the participants was 65.5 years (SD = 11.8), with ages ranging from 36 to 95 years. Most participants (95.5 %) were not mental health patients while 4.5 % were treated for a minor disorder. A total of 69.6 % of the participants had an annual income of 18,000€ or less per year. The indicated use of BZD was 54.7 % for insomnia, 23.9 % for anxiety and 10.7 % for both. The participants had been using BZD for an average of 4.1 years (SD = 4.9), with the duration of use ranging from 4 months to 40 years.

Approximately one-quarter of the participants (24.7 %) had no comorbidities. The majority of patients (43.2 %) had mild comorbidities (ranging from 1 to 2), whereas 23.9 % of the participants fell into the moderate comorbidity category (3 to 4). A smaller portion of participants (8.2 %) had severe comorbidities, with scores of 5 or higher (Table 1).

**Table 1:** Baseline characteristics.

	Participants (*n* = 243)
Male sex, n (%)	74 (30.5)
Age,year (SD)	65.5 (11.8)
Treated by mental minor disorder, n (%)	11 (4.5)
Income ≤18,000 €/year, n (%)	169 (69.6)
**Charlson index, n (%)** • No comorbidities • Mild comorbidity • Moderate comorbidity • Severe comorbidity	60 (24.7) 105 (43.2) 58 (23.9) 20 (8.2)
**Indication for use, n (%)** • Anxiety • Insomnia • Anxiety and insomnia • Others	58 (23.9) 133 (54.7) 26 (10.7) 9 (3.7)
Duration of BZD use, years (SD)	4.1 (5.4)

N: number of patients; SD: standard deviation.

### 3.2 Benzodiazepine discontinuation

**Fig. 2:**
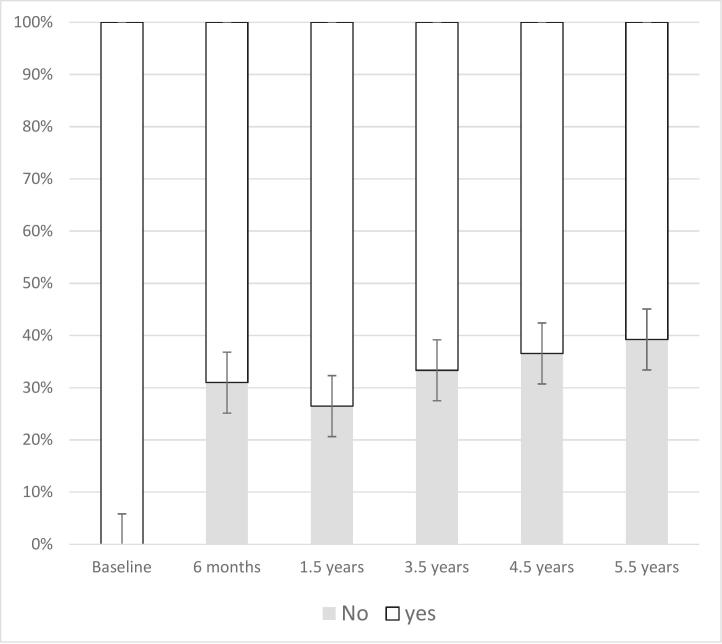
Number and percentage of participants with benzodiazepines use over the time.

We observed a gradual but steady increase in the discontinuation of BZD use over time (Fig. 2). The discontinuation rate rose to 31.3 % at 6 months after intervention, 28 % at 1.5 years, 35 % at 3.5 years, 37.9 % at 4.5 years, and finally reached 40.7 % by the 5.5-year mark.

### 3.3 Changes in risk perceptions and attitudes toward benzodiazepine use

At baseline, the majority of participants (79.4 %) considered BZD to be mild and safe for long-term use. However, after 6 months of intervention, this perception significantly decreased, with only 45.7 % of the participants maintaining this belief (*p* = 0.034) (Table 2).

**Table 2:** Change in risk perception and attitudes.

Variables		Baseline n (%)	6 months n (%)	*p* value⁎
Consider BZD is mild and safe at long-term	No	47 (20.6)	132 (54.3)	0.034
	Yes	181 (79.4)	111 (45.7)	
Consider Anxiety or sleep worse if quit	No	67 (29.1)	89 (36.3)	0.014
	Yes	163 (70.9)	154 (63.4)	
Have taken more than 4 weeks continuously	No	36 (15.5)	96 (39.5)	<0.001
	Yes	197 (85.5)	147 (60.5)	
Previously attempts to cease	No	167 (68.7)	63 (25.9)	<0.001
	Yes	75 (31.3)	180 (74.1)	

⁎ McNemar test.

Before the intervention, 70.9 % of the participants believed that their anxiety or sleep would worsen if they quit BZD. This concern persisted but decreased slightly to 63.4 % at 6 months (*p* = 0.014).

At baseline, 85.5 % of participants reported continuous use of benzodiazepines (BZD) over the previous four weeks. Six months after the intervention, this continuous use significantly decreased to 64.6 %, indicating a shift toward more intermittent use (*p* < 0.001).

Finally, there was a substantial increase in the proportion of participants who attempted to cease previously BZD use, from 31.3 % at baseline to 74.1 % at 6 months (p < 0.001).

### 3.4 Changes in health status

**Fig. 3:**
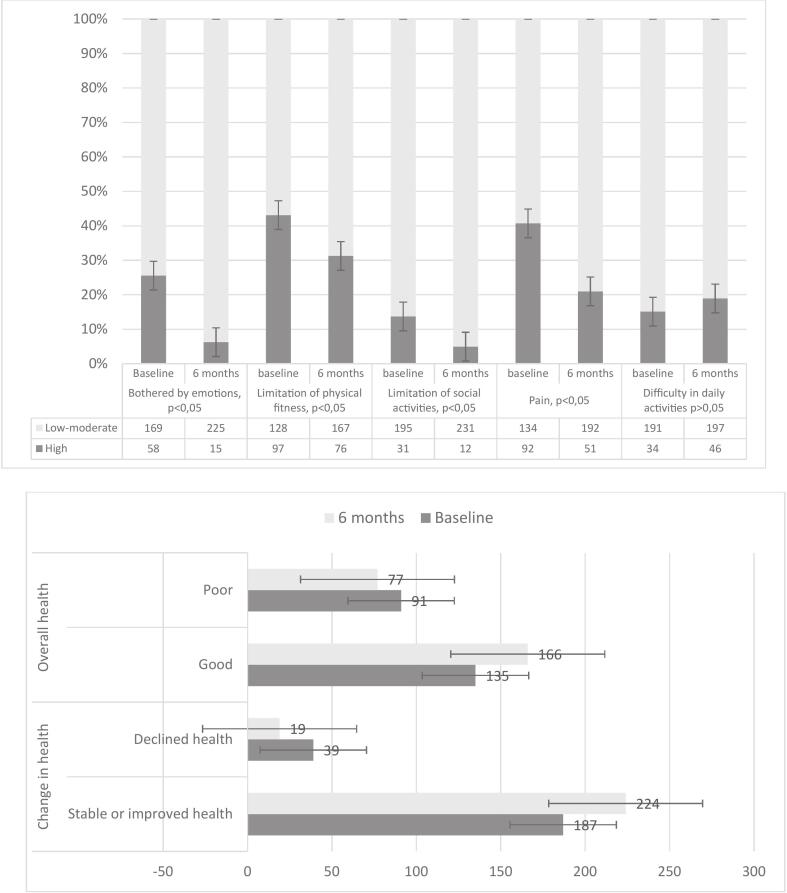
Quality of life (WONCA-COOP) at baseline and 6 months after intervention. McNemar test compared with baseline.

Fig. 3 illustrates changes in participants' quality of life indicators between baseline and 6 months postintervention across seven domains: being bothered by emotions, having limited physical fitness, having limited social activity, experiencing pain, having difficulty performing daily activities, overall health and changes in health, over time.

At baseline, 58 participants (25 %) reported highly bothered by emotions, which decreased significantly to 15 participants (6 %) at the 6-month follow-up (*p* < 0.05). Similarly, the number of participants experiencing high limitations in physical fitness decreased from 97 participants (43 %) to 76 (31 %) after 6 months (p < 0.05). Additionally, reported high limitations in social activity by 31 participants (13 %) at baseline, which decreased to 12 participants (5 %) at 6 months (p < 0.05). In terms of pain, 92 participants (41 %) reported high levels of pain at baseline, but this number dropped to 51 participants (21 %) at the 6-month mark (p < 0.05). In contrast, high difficulties in daily activities increased slightly but non-significantly, from 34 participants (15 %) at baseline to 46 participants (19 %) at 6 months (*p* > 0.05).

Changes in overall health and health stability after six months of the intervention aimed at reducing BZD use, were as follows. At baseline, 135 participants (60 %) reported good overall health, whereas 91 participants (40 %) reported poor health. After six months, the number of participants reporting good health increased to 166 (68 %), with a corresponding decrease in those reporting poor health to 77 (32 %). Although there was an observable improvement in overall health status, this change was not statistically significant (*p* > 0.05).

Similarly, for changes in health over time, 187 participants (83 %) reported stable or improved health at baseline, increasing to 224 participants (92 %) at the six-month follow-up. Those who reported a decline in health decreased from 39 participants (17 %) at baseline to 19 participants (8 %) at six months. While the trend suggests improvements in health stability, these changes also did not reach statistical significance (p > 0.05).

### 3.5 Associations between changes in the risk perceptions, attitudes and health status of patients and BZD use after 5.5 years of intervention

The factors associated with BZD use after 5.5 years were: considerer BZD to be mild and safe for long-term use, anxiety or sleep worsening if BZD were discontinued, continued use 4 weeks, and no previous attempts to quit. However, changes in health status did not have a statistically significant effect on long-term BDZ discontinuation. The logistic regression models exhibited acceptable goodness-of-fit, with AIC-Akaike Information Criterion- values improving from an average of 450.3 (unadjusted models) to 423.7 (adjusted models including baseline covariates, socio-economic and educational level, and relevant confounders). Additionally, likelihood ratio tests comparing nested models confirmed significant improvements in model fit (*p* < 0.05).

The participants who considered BZD to be mild and safe for long-term use at six months after the intervention were twice as likely to continue BZD consumption (OR = 2.03, 95 % CI: 1.58–2.61, *p* < 0.001). Similarly, those who believed that their anxiety or sleep would worsen if they stopped using BZD were 4.67 times more likely to continue using BZD (95 % CI: 3.57–6.13, p < 0.001).

With respect to use patterns, participants who reported having taken BZD for more than 4 weeks continuously (not intermittent use of BZD) were significantly more likely to continue using BZD than those with more not consistent use (OR = 4.9, 95 % CI: 3.69–6.45, p < 0.001).

Participants without previous cessation attempts were more likely to continue BZD use (OR = 2.09, 95 % CI: 1.53–2.84, p < 0.001) (Table 3).

**Table 3:** Risk perception, attitudes and health change at 6 months post-intervention and association with BZD use at follow-up.

Variables	Adjusted Odds-ratio⁎	Std. Err.	CI 95 %	P value
Consider BZD is mild and safe at long-term	2.03	0.26	1.58–2.61	<0.001
Consider Anxiety or sleep worse if quit	4.67	0.64	3.57–6.13	<0.001
Have taken more than 4 weeks continuously (Not intermittent use)	4.9	0.69	3.69–6.45	<0.001
Not previously attempts to cease	2.09	0.33	1.53–2.84	<0.001
Change in health at 6 months	1.06	0.06	0.97–1.24	0.125

⁎ Adjusted odds ratios for continued BZD use, controlling for mental health status, sex, centre, age, income, and comorbidity. Period 2 (4.5 years) excluded due to collinearity. p < 0.05 considered statistically significant.

### 3.6 Changes in quality of life

Quality of life (COOP/WONCA) was assessed in the same participants at baseline and 6 months (pre–post). Improvements represent within-person change across domains (emotional symptoms, physical fitness, social activity, pain, daily activities, overall health, and health change over time). Overall, several domains improved at 6 months (Fig. 3), and no deterioration was observed. In adjusted models, 6-month QoL change was not significantly associated with sustained BZD discontinuation at 5.5 years (adjusted OR 1.06, 95 % CI 0.97–1.24; *p* = 0.125). No adverse effects or harms were reported during the intervention period.

## 4 Discussion

The findings from this study underscore the long-term effectiveness of the *BenzoStopJuntos* intervention in patient attitudes and beliefs regarding BZD use at the six-month mark, promoting sustained BZD discontinuation over a five-and-a-half-year period. This is consistent with results of brief interventions at 10 years follow-up^30^.

This outcome supports the value of direct-to-patient interventions in BZD deprescribing among older adults and reinforces patient education as a key strategy to shift long-standing attitudes and behaviors. Although most previous studies have focused on provider-led educational strategies aimed at improving prescribing practices,^31^^,^^32^ growing evidence supports the effectiveness of patient-directed interventions. The EMPOWER trial^19^ and the D-PRESCRIBE^20^ trial demonstrated that directly educating older adults about the risks of benzodiazepines significantly reduces inappropriate use and empowers patients to initiate deprescribing conversations with healthcare providers. Our findings align with these results and reinforce the value of direct-to-patient education as a key component of sustainable deprescribing strategies. By equipping patients with accessible information and behavioral tools, these interventions help overcome psychological barriers and promote shared decision-making, ultimately supporting long-term medication discontinuation.

We identified two key factors that strongly predict BZD deprescribing success: concerns about worsening anxiety or sleep if BZD are discontinued and continuous use (longer than four weeks), as opposed to intermittent use. The participants who worried about worsened anxiety or sleep were 4.7 times more likely to continue BZD use. At baseline, 70.9 % of participants feared that stopping BZD would negatively impact their sleep or anxiety; by six months, this percentage decreased to 63.4 %, yet participants who retained this belief were five times more likely to continue using BZD. This highlights the need for interventions that directly target these fears, as they create perceived barriers to discontinuation that often reinforce dependency.^33^, ^34^, ^35^

Similarly, continuous BZD usage was a strong predictor of ongoing dependence, with nearly a 5-fold increased likelihood of continued use among those using BZD consistently rather than intermittently. This underscores the need for future interventions to address both psychological and behavioral components in BZD discontinuation. Encouraging intermittent use and early cessation attempts could be essential to achieving long-term discontinuation success.

At baseline, 79.4 % of the participants who believed that BZD were mild and safe for long-term use, a belief that greatly hindered deprescribing efforts. After the intervention, this percentage decreased to 45.7 %, illustrating the effectiveness of patient education in shifting attitudes toward BZD use. The participants who believed that BZD were safe were nearly twice as likely to continue BZD use. This finding is consistent with those of previous studies, which have shown that long-term BZD use often underestimates the risks associated with chronic use, such as dependency, cognitive decline, and increased risk of falls, particularly in older populations.^36^^,^^37^ By challenging these beliefs through education and personalized tapering plans, the *BenzoStopJuntos* intervention enabled many participants to reconsider the necessity of continued BZD use.

Our study suggests that participants who experienced changes in risk and attitudes as a result of the intervention may have undergone cognitive dissonance, serving as the underlying mechanism for heightened risk perceptions. Those with increased risk perceptions reported higher self-efficacy in gradually reducing BZD use and a strong intention to engage in preventive health behaviors by discussing medication safety with a healthcare professional.^38^

The intervention's success in reducing these fears can be attributed to the implementation of alternative treatment strategies, including cognitive-behavioral therapy (CBT) delivered in self-guided, individual, or group formats, specifically designed to address anxiety and insomnia management,^39^^,^^40^ as well as other non-pharmacological approaches. Studies have shown that providing such alternatives can significantly reduce reliance on BZD.^41^^,^^42^

The evidence also indicates that BZD deprescription is associated with subtle cognitive advantages, improvements in daytime activity,^43^ social relationships, physical and psychological health as in our study ^41^.

A major strength of this study is the multidisciplinary nature of the *BenzoStopJuntos* intervention, which involved coordinated efforts among general practitioner, social workers, pharmacists, and nurses. This collaborative approach allowed for consistent monitoring and support throughout the tapering process, contributing to the intervention's success. All these elements were key to achieving the objectives, resolving doubts and effective collaboration between the different agents.^25^^,^^44^ Furthermore, the five-and-a-half-year follow-up period provided a robust assessment of the long-term impact of the intervention, revealing the gradual but sustained increase in BZD discontinuation over time.

However, several limitations should be considered. While the quasi-experimental design lacks a concurrent control group, this approach allows for a more naturalistic evaluation of the intervention's long-term impact in real-world settings. Nevertheless, incorporating a comparison group through cluster randomization or retrospective analysis could further enhance the study's validity by providing more rigorous causal inference. Additionally, attitudes and perceptions toward BZD use were self-reported, which may introduce response bias, though the consistency of the findings with prior research suggests that these self-reports were reliable.^45^ Furthermore, the study did not capture BZD prescriptions obtained outside the public healthcare system, which may have led to an underestimation of continued BZD use. The authors believe that this under-reporting should be negligible, given the implementation of the public health system and the characteristics of the majority of the population, i.e. pensioners and mostly free prescriptions. Considering that, after five and a half years of follow-up, changes in risk perceptions and attitudes are associated with the deprescription of BZD reinforcing these essential component elements. Because clinicians delivered the intervention, some performance bias is possible; this risk was minimised by using external assessors at 6 months and objective dispensing records for long-term outcomes. Finally, although the optional medical consultation and referral to the socio-educational group were not core components of the intervention, data collected indicate that only a small proportion of participants used these additional resources. This suggests that the core intervention—focused on changing risk perception and attitudes—was sufficient to achieve sustained impact. Moreover, the lack of increased healthcare utilization among participants supports the idea that these optional components did not introduce significant bias into the results.

Direct-to-patient strategies are uniquely positioned to address both patient- and provider-related barriers to deprescribing, as highlighted in studies that evaluate similar deprescribing strategies.^32^^,^^35^ These interventions effectively circumvent “prescriber inertia,” where time constraints or other factors hinder provider engagement in deprescribing. When patients are educated about medication risks, they are more inclined to initiate conversations with providers, resulting in shared decision-making that supports deprescribing.^31^^,^^35^ This strategy is crucial for sustainability, as patients who are empowered to discuss their treatment options tend to achieve higher rates of medication discontinuation^33^.

## 5 Conclusions

The BenzoStopJuntos intervention demonstrated significant long-term effectiveness in promoting BZD discontinuation, driven primarily by shifts in patient risk perceptions and attitudes. Key predictors of successful discontinuation were the beliefs about BZD and intermittent BZD use. Participants who viewed BZD as safe for prolonged use were twice as likely to continue usage, while those who feared worsened anxiety or sleep were nearly five times more likely to persist in BZD consumption. Intermittent BZD use prior and attempts to cease was a significant predictor of discontinuation success, suggesting that promoting intermittent use patterns may support gradual discontinuation efforts.

Importantly, the intervention's success underscores the value of a multidisciplinary, patient-centered approach that combines education, behavioral strategies, and alternative therapies. These findings have substantial implications for public health strategies aimed at reducing BZD dependence, particularly in high-risk populations such as older adults. By addressing psychological barriers and offering non-pharmacological alternatives, the *BenzoStopJuntos* model provides a replicable framework for deprescribing interventions in primary care settings.

## CRediT authorship contribution statement

**Ingrid Ferrer López:** Writing – review & editing, Validation, Funding acquisition, Conceptualization. **Antonio Olry de Labry-Lima:** Writing – review & editing, Validation, Investigation. **Alicia Gutiérrez-Valencia:** Writing – review & editing, Validation, Investigation. **Encarnación García Bermúdez:** Writing – original draft, Investigation. **Francisco Atienza Martín:** Writing – review & editing, Investigation. **Amalia García-Delgado Morente:** Writing – review & editing, Investigation. **María Dolores Murillo Fernández:** Writing – review & editing, Investigation. **Yolanda Sánchez Cañete:** Writing – review & editing, Investigation. **Clara Bermúdez-Tamayo:** Writing – original draft, Methodology, Investigation, Conceptualization.

## Consent for publication

Not applicable.

## Ethics approval and consent to participate

The study was approved by the Health Research Ethics Board of Hospitals Virgen Macarena-Virgen del Rocío (Approval Reference: IFLBZD16) and conducted in accordance with the Declaration of Helsinki and Good Clinical Practice (GCP) guidelines. All participants provided written informed consent after being informed of their voluntary participation and right to withdraw at any time without affecting their healthcare. Potential risks and benefits were explained, and the principal investigator's contact details were provided. Data collection adhered to confidentiality and privacy regulations, including General Data Protection Regulation (GDPR) standards, with anonymized patient and healthcare provider data ensuring non-identifiability. All study procedures followed ethical guidelines and relevant institutional and national regulations.

## Funding sources

This study without initial financing was supported by several awards that contributed to its funding over time, including Best Communication Award at the 4th National Medical & Pharmaceutical Congress, organized by SEMERGEN (Spanish Society of Primary Care Physicians) and SEFAC (Spanish Society of Family and Community Pharmacy), 2022. Best Communication Award at the 2nd National Medical-Pharmaceutical Congress by SEMERGEN and SEFAC, 2020, and Best Research Project Award at the XII Andalusian Congress of Primary Care Physicians by SEMERGEN, 2017.

## Declaration of competing interest

The authors declare that they have no known competing financial interests or personal relationships that could have appeared to influence the work reported in this paper.

## Data Availability

The datasets generated and analysed during the current study are available from the corresponding author on reasonable request.
